# Sortase-encoding genes, *srtA* and *srtC*, mediate *Enterococcus faecalis* OG1RF persistence in the *Helicoverpa zea* gastrointestinal tract

**DOI:** 10.3389/fmicb.2024.1322303

**Published:** 2024-03-18

**Authors:** Jerreme J. Jackson, Samantha Heyer, Geneva Bell

**Affiliations:** Department of Biology, University of Northern Iowa, Cedar Falls, IA, United States

**Keywords:** *Helicoverpa zea*, *Enterococcus faecalis*, biofilm, sortase, persistence

## Abstract

*Enterococcus faecalis* is a commensal and opportunistic pathogen in the gastrointestinal (GI) tract of mammals and insects. To investigate mechanisms of bacterial persistence in the gastrointestinal tract (GIT), we developed a non-destructive sampling model using *Helicoverpa zea*, a destructive agricultural pest, as host to study the role of bacterial sortase enzymes in mitigating persistence in the gastrointestinal tract. *E. faecalis* OG1RF Δ*srtA* and *E. faecalis* OG1RF Δ*srtC*, isogenic *E. faecalis* OG1RF sortase mutants grew similarly under planktonic growth conditions relative to a streptomycin-resistant *E. faecalis* OG1RFS WT *in vitro* but displayed impaired biofilm formation under, both, physiological and alkaline conditions. In the *H. zea* GI model, both mutants displayed impaired persistence relative to the WT. This represents one of the initial reports in which a non-destructive insect model has been used to characterize mechanisms of bacterial persistence in the Lepidopteran midgut and, furthermore, sheds light on new molecular mechanisms employed by diverse microorganisms to associate with invertebrate hosts.

## 1 Introduction

Nosocomial infections represent the most frequent negative consequences of healthcare delivery. The World Health Organization estimates that the prevalence rate of hospital acquired infections in the United States to be at 5%. Moreover, an alarming number of patients in developed countries will acquire at least one nosocomial infection, leading to increased mortality, morbidity, and a decreased quality of life (Raoofi et al., 2023). The animal intestinal microbiome is recognized as a natural reservoir for many nosocomial pathogens, including Enterobacteriaceae, Enterococcus, Candida, and Clostridia species (Kim et al., 2017).

*Enterococcus faecalis* is a gram-positive facultative anaerobe commonly identified among the diverse microbial species in the gastrointestinal (GI) tract of vertebrates and insects (Martin and Mundt, 1972; Krawczyk et al., 2021). Studies have shown *E. faecalis* to be one of the earliest commensal colonizers of the naïve mammalian gastrointestinal tract (GIT) (Noble, 1978; Al-Balawi and Morsy, 2020). At the genus level, *Enterococcus* spp. were detected in greater than 70 % of screened lepidopteran species [reviewed in Paniagua Voirol et al. (2018)]. The prevalent use of antibiotic treatment in clinical environments, the intrinsic resistance of *E. faecalis* to broad spectrum antibiotics, and the horizonal gene transfer of resistance-associated genetic elements between *E. faecalis* and other clinically significant bacteria have contributed to its emergence as the leading cause of nosocomial enterococcal infections (Hollenbeck and Rice, 2012; Fiore et al., 2019; Garcia-Solache and Rice, 2019). While urinary tract infections have, historically, been the most frequently reported complications associated with *E. faecalis*, it has also been linked to bacteremia, infective endocarditis, meningitis, and endodontic infections (Breton et al., 2002; National Nosocomial Infections Surveillance, 2004; Nallapareddy et al., 2006; Fisher and Phillips, 2009; Khan et al., 2018; Gaeta et al., 2023).

Insects belonging to the order Lepidoptera, such as *Helicoverpa zea* (corn earworm), have historically been used as infection models to elucidate the mechanisms of crystalline and vegetative insecticidal proteins produced by the entomopathogenic bacterium, *Bacillus thuringiensis* (Schnepf et al., 1998; Jouzani et al., 2017; Syed et al., 2020). However, over the last two decades, lepidopteran larvae have emerged as complementary tools to study enterococcal virulence factors and host innate immune defenses (Kanost et al., 2004; Broderick et al., 2009; Mason et al., 2011). Morphologically, the lepidopteran GIT is simple relative to other insect orders, and ingested substances experience retention times of approximately two hours (Brinkmann and Tebbe, 2007; Engel and Moran, 2013). The luminal pH in the midgut region of the GIT ranges from 9–12 in many lepidopteran species and poses challenges to microorganisms better adapted to neutral or slightly acidic environments (Kakinuma, 1987; Appel and Martin, 1990; Kakinuma and Igarashi, 1990; Dow, 1992). It is plausible to expect host-derived factors such as GIT alkalinity and intestinal peristalsis driving the continuous turnover of luminal contents affect many aspects of commensal physiology and the ability to a stably colonize.

Pili are hair-like proteinaceous extrusions that extend from the cell wall into the cytoplasm surrounding gram-negative and gram-positive bacteria. In addition to their role in bacterial conjugation, research has shown that pili also mediate binding to extracellular matrix proteins and glycoproteins in the mucosal layer of specific host tissues and virulence (Vimal et al., 2000; Proft and Baker, 2009; Nallapareddy et al., 2011; Sillanpaa et al., 2013; Flores-Mireles et al., 2014; Montealegre et al., 2016). Membrane-anchored transpeptidases called sortases recognize a conserved LPXTG-motif on substrates and covalently attach them to peptidoglycan in the cell wall of gram-positive bacteria (Marraffini et al., 2006). The *E. faecalis* genome encodes two sortases: the housekeeping sortase, *srtA*, and the biofilm- and pilus-associated sortase, *srtC* (referred to elsewhere as *bps*), which catalyzes the assembly of the endocarditis- and biofilm-associated pilin subunits (Sillanpaa et al., 2013). Currently, neither the role of cell surface proteins nor pili in bacterial persistence in the lepidopteran GIT has been characterized.

In this report we compared wild-type (WT) *E. faecalis* OG1RF to two isogenic mutants harboring deletions of either the housekeeping sortase, *srtA*, or *srtC*, the biofilm- and pilus-associated sortase, during planktonic growth and in biofilm assays under alkaline conditions. Using a *H. zea* caterpillar model, we also tested the effects of the sortase deletions on *E. faecalis*-induced frass production and colonization of the inherently alkaline GIT.

## 2 Materials and methods

### 2.1 Insects and rearing conditions

*H. zea* eggs and general-purpose lepidopteran diet were purchased from Frontier Agricultural Sciences (Newark, DE). After hatching, larvae were maintained in 1.25 oz. plastic cups (Frontier, Newark, DE) on diet under standard rearing conditions including a photoperiod of 16: 8 (L:D), 25°C, and 65% RH in a Percival Intellus Environmental Controller (Percival, Perry, IA).

### 2.2 Media preparation and bacterial strains

Bacterial strains were incubated in Brain Heart Infusion (BHI) broth (RPMI, ThermoFisher, Waltham, MA) prepared according to the manufacturer’s instructions or yeast peptone base media supplemented as follows. Briefly, yeast peptone base (YP) medium was supplemented with glucose (2% [wt./vol.], YPD), sucrose (2% [wt./vol.], YPS), lactose (2% [wt./vol.], YPL), or maltose (2% [wt./vol.], YPM), adjusted to the desired pH using an Accumet Benchtop pH Meter (ThermoFisher, Waltham, MA), then filtered through a 0.22 μm mixed cellulose ester membrane (MCE) (ThermoFisher, Waltham, MA) inside a Purifier Logic+ Class II Biosafety Cabinet (BSC) (Labconco, Fort Scott, KS). *Enterococcus faecalis* OG1RF is a rifampicin and fusidic acid resistant derivative of *E. faecalis* OG1 which was first isolated from a human oral cavity (Gold et al., 1975; Dunny et al., 1978). Bacteria strains used in this study are listed in Supplementary Table S1.

### 2.3 Isolation of streptomycin-resistant *Enterococcus faecalis* OG1RF clones

A single *E. faecalis* OG1RF colony was inoculated into 5 mL of BHI containing 50 μg ml^−1^ rifampicin and incubated overnight with shaking at 37°C in a MaxQ 4,000 orbital shaker (ThermoFisher, Waltham, MA). The stationary phase culture was diluted (1:10 [vol./vol.]) into fresh media and, and the absorbance at 600 nm was monitored using a Biomate™ 3 Series spectrophotometer (ThermoFisher, Waltham, MA) until mid-log phase growth. Fifty microliters of the mid-log phase *E. faecalis* OG1RF culture was plated using autoclaved borosilicate glass beads (ThermoFisher, Waltham, MA) on brain heart infusion agar plates containing 50 μg ml^−1^ rifampicin and 100 μg ml^−1^ streptomycin and incubated overnight at 37°C in a Steri-Cult CO_2_ incubator (ThermoFisher, Waltham, MA) for 24 h.

### 2.4 Colony PCR and purification of DNA fragments containing *rpsL*

Streptomycin-resistant *E. faecalis* OG1RF colonies appearing within 24 h after plating were selected at random for colony pcr (cPCR). Briefly, each colony was touched gently with a sterile toothpick, transferred to an autoclaved 1.5 mL microcentrifuge tube (ThermoFisher, Waltham, MA) containing 100 μL deionized distilled water (ddH_2_O), and pipetted several times to mix. Suspended bacteria were lysed by boiling for 5 min in a digital dry bath (Corning, Glendale AZ) and centrifuged at room temperature for 1 min at 13,300 RPM in an Eppendorf 5430 microcentrifuge (Eppendorf, Framingham, MA) to separate soluble genomic DNA (gDNA) from cell debris. One microliter of supernatant from each tube was used as template DNA in a single pcr in the GoTaq^®^ Green Master Mix (Promega Corp, Madison, WI) according to the manufacturer’s instructions in a MyCycler™ Thermal Cycler (BioRad, Hercules, CA). Referencing the *E. faecalis* OG1RF complete genome sequence (accession number CP002621.1) available from the National Center for Biotechnology Information (NCBI), custom primers (forward 5′- ACCACCTGGATGTGTGGAAC-3′, reverse 5′- CGAGGCATCCGTAACTCCTC-3′) were designed to amplify a 530 bp region including the gene encoding the ribosomal S12 protein (*rpsL*). The PCR conditions were as follows: 95°C for 2 min, 35 cycles of 95°C for 30 s, 55°C for 32 s, and 72°C for 25 s, and a final extension at 72°C for 5 min. Amplicon sizes were confirmed by electrophoresing 2 μL of each cPCR in a agarose gel (1% [wt./vol.]) as described previously (Green et al., 2012). The remaining volume of each cPCR was purified using the GeneJET PCR Purification Kit (ThermoFisher, Waltham, MA) according to the manufacturer’s instructions and quantified using a NanoPhotometer^®^ NP80 (Implen, Westlake Village, CA).

### 2.5 Sequencing and analysis

Purified cPCR amplicons were shipped to the Iowa State University DNA Facility for sequencing using the Sanger Method, and sequences were aligned using Clustal Omega (Sievers et al., 2011). Custom primers (forward 5’-TACGGATGTTAATTGGTTAATC-3′, reverse 5′- AATTCGAAATCCTGCAAAAC -3′) were designed to sequence the 414 bp *rpsL* coding region.

### 2.6 *In vitro* streptomycin resistance

To test *E. faecalis* OG1RF and *E. faecalis* OG1RFS for levels of streptomycin resistance, strains were incubated overnight in 5 mL YPD containing 50 μg ml^−1^ rifampicin with shaking at 37°C in a MaxQ 4,000 orbital shaker (ThermoFisher, Waltham, MA). Five microliters of stationary phase cultures were added to 995 μL of YPD (1:200 dilution, OD_600_ = 0.005) into fresh YPD containing 50 μg ml^−1^ rifampicin, and either 100 μg ml^−1^, 1 mg ml^−^1, or 10 μg ml^−1^ streptomycin. Two hundred microliters of each strain in all treatment conditions were transferred in triplicate to wells of a 96-well flat-bottom microtiter plate (Falcon, Waltham, MA). Plates were incubated in a BioTek Synergy HT plate microplate reader at 37°C with continuous medium-level shaking, and the optical density at 600 nm (OD_600_) was read every 15 min. The experiment was repeated in triplicate.

### 2.7 *In vitro* carbon source utilization and streptomycin resistance

Carbon source utilization by *E. faecalis* OG1RFS, *E. faecalis* OG1RF Δ*srtA*, and *E. faecalis* OG1RF Δ*srtC* was tested as follows: Strains were incubated overnight in 5 mL YPD containing 50 μg ml^−1^ rifampicin with shaking at 37°C as previously described here. Five microliters of stationary phase cultures were added to 995 μL (1:200 dilution, OD_600_ = 0.005) of fresh YPD, YPS, YPL or YPS containing 50 μg ml^−1^ rifampicin and 100 μg ml^−1^ streptomycin (*E. faecalis* OG1RFS) or only 50 μg ml^−1^ rifampicin (*E. faecalis* OG1RF Δ*srtA* and Δ*srtC*), and 200 μL was transferred in triplicate to wells of a 96-well flat-bottom microtiter plate. Plates were incubated in a BioTek Synergy HT plate microplate reader at 37°C with continuous medium-level shaking, and the optical density at 600 nm (OD_600_) was read every 15 min. The time for all strains incubated in YPD, YPS, YPL, and YPM at pH 7.4 and pH 10 to cross the OD_600_ = 0.4 threshold was used to indicate entry into mid-log phase growth. The experiment was repeated in triplicate.

### 2.8 Association and quantification of *Enterococcus faecalis* in the *Helicoverpa zea* gastrointestinal tract

*H. zea* eggs in 2 in.^2^ cheese cloth pieces were surface sterilized by submerging for 5 min in 100 μL of a chlorination solution (0.6% Clorox [vol./vol.], 0.1% Triton X-100 [vol./vol.]) inside an autoclaved 250 mL polysulfone bottle top filter (ThermoFisher, Waltham, MA) with intermittent swirling. Next, the chlorination solution was removed by filtration through a 0.22 μm MCE membrane. Removal of the chlorination solution was followed by two 5 min rinses in 100 mL of autoclaved ddH_2_O. Cheesecloths were transferred to a biosafety cabinet and dried on Kimwipes (ThermoFisher, Waltham, MA). After drying, sterilized eggs were incubated at 37°C inside ethanol cleansed and UV-treated 8 oz. deli cups (Bare, Lancaster, PA). Upon hatching, neonates were maintained on general purpose lepidopteran diet in 1.25 oz. plastic cups. After visual signs of molting to the third instar, larvae were transferred to empty 1.25 oz. cups and starved overnight. On the same day, *E. faecalis* OG1RFS, *E. faecalis* OG1RF Δ*srtA*, and *E. faecalis* OG1RF Δ*srtC* colonies were inoculated in 5 mL BHI with the appropriate antibiotics and incubated overnight as described previously. On the next day (association day), 1 mL of each stationary phase culture was pelleted by centrifugation at room temperature for 5 min at 13,300 RPM and washed twice in sterile phosphate-buffered saline (PBS, 137 mM NaCl, 2.7 mM KCl, 10 mM Na_2_HPO_4_, 1.8 mM KH_2_PO_4_) pH 7.4. Pellets were resuspended in 10 mL PBS (~10^8^ CFU ml^−1^), and 100 μL of each strain was pipetted onto the surface of general-purpose Lepidopteran diet amended with 50 μg ml^−1^ rifampicin that was previously dispended in the wells of a bioassay tray (Frontier, Newark, DE) and gently swirled to ensure even coating. After drying (~ 4 h), three larvae were placed on diet coated with each bacteria strain, covered with ventilated adhesive seals (Frontier, Newark, DE), and incubated under the previously described standard rearing conditions. Each day post-association, larvae were carefully transferred to fresh diet amended with 50 μg ml^−1^ rifampicin, without disturbing the feces (frass), and returned to the incubator. Next, sterile blunt-end forceps were used to transfer frass samples from each larva into separate autoclaved 2 mL microcentrifuge tubes (ThermoFisher, Waltham, MA). Frass samples were weighed using an OHAUS Analytical Balance (OHAUS, Parsippany, NJ), diluted in PBS (1:10 [wt./vol.]), and vortexed every 15 min for one hour to liberate bacteria. The soluble fraction of each homogenate was serially diluted in sterile PBS (1:10 [vol./vol.]) and 100 μL was spread using autoclaved borosilicate glass beads (ThermoFisher, Waltham, MA) on BHI agar plates containing appropriate antibiotics. Plates were incubated statically at 37°C for 24–48 h before colonies were counted. Viable counts of each strain were log transformed and reported as the average log_10_ CFU per gram of frass. The experiment was repeated in triplicate.

### 2.9 *Enterococcus faecalis* microtiter plate biofilm assays

To test the effects of decreased carbohydrate resources on biofilm growth at physiological (pH 7.4) and alkaline (pH 10) conditions, *E. faecalis* OG1RFS, *E. faecalis* Δ*srtA*, and *E. faecalis* Δ*srtC* were inoculated into 5 mL YPD containing 50 μg ml^−1^ rifampicin and incubated overnight with shaking at 37°C as previously described. Serial dilutions of YPD, YPS, YPL, and YPM at pH 7.4 and 10 were prepared by diluting each with a YP medium (1:3 [vol./vol.]) of the same pH. Five microliters of stationary phase cultures were added to 995 μL (1:200 dilution, OD_600_ = 0.005) of serially diluted YPD, YPS, YPL, and YPM media containing 50 μg ml^−1^ rifampicin and 100 μg ml^−1^ streptomycin (*E. faecalis* OG1RFS) or only 50 μg ml^−1^ rifampicin (*E. faecalis* OG1RF Δ*srtA* and Δ*srtC*), and 200 μL was transferred in triplicate to wells of a 96-well flat-bottom microtiter plate. Plates were incubated statically at 37°C for 24 h. Adherent cells were stained and quantified following a previously described method (O’toole, 2011). For staining, planktonic and non-adherent cells were removed by inverting the microtiter plates and gently shaking out the media. Next, the microtiter plates were submerged in ddH_2_O and inverted to rinse away unattached cells and residual media. This step was performed a minimum of four times. After rinsing, plates were gently blotted on paper towels and left inverted on fresh paper towels to air dry for 5–10 min at room temperature. Adherent cells were stained for 15 min by adding 125 μL crystal violet (0.1% [wt./vol.]) to all wells using a multi-channel pipet. Crystal violet stain was removed following the rinse steps described above, and microtiter plates were inverted on fresh paper towels and allowed to air dry overnight. The next day, crystal violet absorbed by cell wall peptidoglycan in adherent cells was solubilized by adding 125 μL of glacial acetic acid (30% [vol./vol.]) to all wells and incubating the microtiter plates at room temperature for 15 min. The solubilized crystal violet in all wells was transferred by pipetting to a new microtiter plate, and the absorbance at 550 nm was read on a Synergy HT microplate reader. The data shown represents the average of data obtained in two independent experiments.

### 2.10 Statistical analysis

Statistically significant differences in the planktonic growth delay of all strains in yeast peptone-based media *in vitro* was determined by a one-way analysis of variance (ANOVA) using Holm-Sidak multiple pairwise comparison tests (*p = 0.05). Bacterial persistence data failed the equal variance (Brown-Forsythe) and normality tests (Shapiro–Wilk) on day two and three, respectively, so we determined significant differences using Welch’s test (**p = 0.05). Statistically significant differences in Biofilm formation data were determined by Welch’s test (**p = 0.05). When biofilm data failed the normality test (Shapiro–Wilk), we determined statistically differences using Mann–Whitney Rank Sum tests (***p = 0.05). SigmaPlot 14.0 (Systat, Palo Alto, CA) was used for all data analyses and the production of graphs. The strength of significance (effect size) was calculated in Microsoft Excel as the absolute value of the difference in the means divided by the pooled standard deviations of two groups, and reported as Cohen’s d value (Cohen, 1988).

## 3 Results

### 3.1 *Enterococcus faecalis* OG1RFS has a single-nucleotide substitution in *rpsL* and is resistant to streptomycin

**Figure 1:**
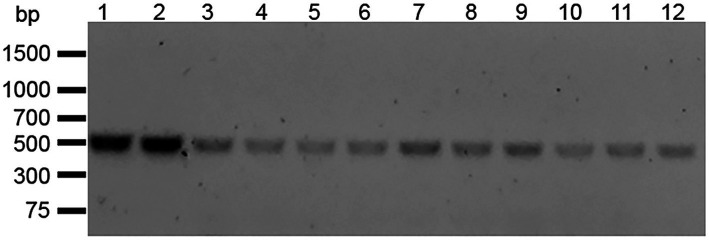
Amplification of the full-length *E. faecalis* OG1RF *rpsL*. Gene-specific forward and reverse primers were designed to amplify *rpsL* including up- and down-stream flanking regions. PCR products were separated by 1% agarose gel electrophoresis. Lane 1: *E. faecalis* OG1RF WT *rpsL.* Lane 2–12: Streptomycin-resistant *E. faecalis* OG1RFS clones 1–11.

**Figure 2:**
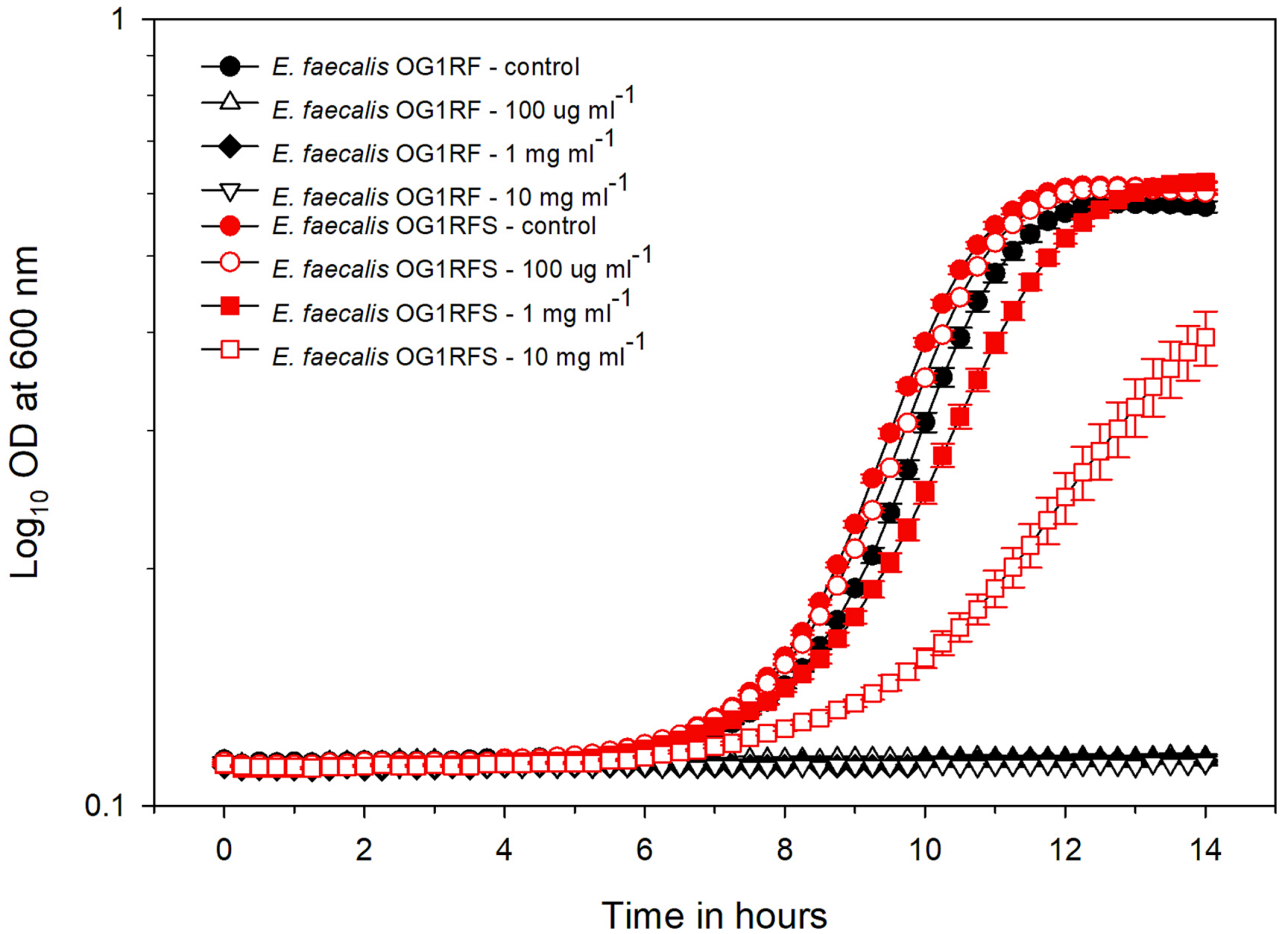
Growth of *E. faecalis* OG1RF WT and an *E. faecalis* OG1RFS WT in YPD medium containing different concentrations of streptomycin. The wild-type and OG1RFS WT were grown in YPD medium with different concentrations of streptomycin for 14 hours. Error bars represent the standard error of the means of OD_600_ at each time point.

We isolated 11 streptomycin-resistant *E. faecalis* OG1RF WT (hereafter referred to as *E. faecalis* OG1RFS WT) clones after spreading mid-log phase cultures on BHI agar containing 50 μg ml^−1^ rifampicin and successfully amplified *rpsL*, which encodes the ribosomal S12 protein, including 50 bp up- and down-stream flanking regions (Figure 1). Sanger sequencing and Clustal Omega alignment indicated that all 11 streptomycin-resistant clones contained a single-nucleotide substitution (167A > G) resulting in a Lys56Arg substitution in the amino acid sequence of RPSL (See clone sequences in Supplementary material). To test the susceptibility of *E. faecalis* OG1RFS to streptomycin, growth was measure in YPD *in vitro* in the presence of increasing streptomycin concentrations. Relative to WT *E. faecalis* OG1RF, which failed to grow in the presence of streptomycin, *E. faecalis* OG1RFS grew under all conditions tested. *E. faecalis* OG1RFS incubated in the presence of 100 μg ml^−1^ streptomycin exhibited a slight delay in reaching exponential growth relative to controls, whereas exponential growth of *E. faecalis* OG1RFS in 1 mg ml^−1^ streptomycin was delayed by approximately 1 h. Exponential growth of *E. faecalis* OG1RFS in 10 mg ml^−1^ streptomycin was delayed by approximately three hours, relative to controls (Figure 2).

### 3.2 *Enterococcus faecalis* OG1RF Δ*srtC* Exhibits delayed growth *in vitro*

**Figure 3:**
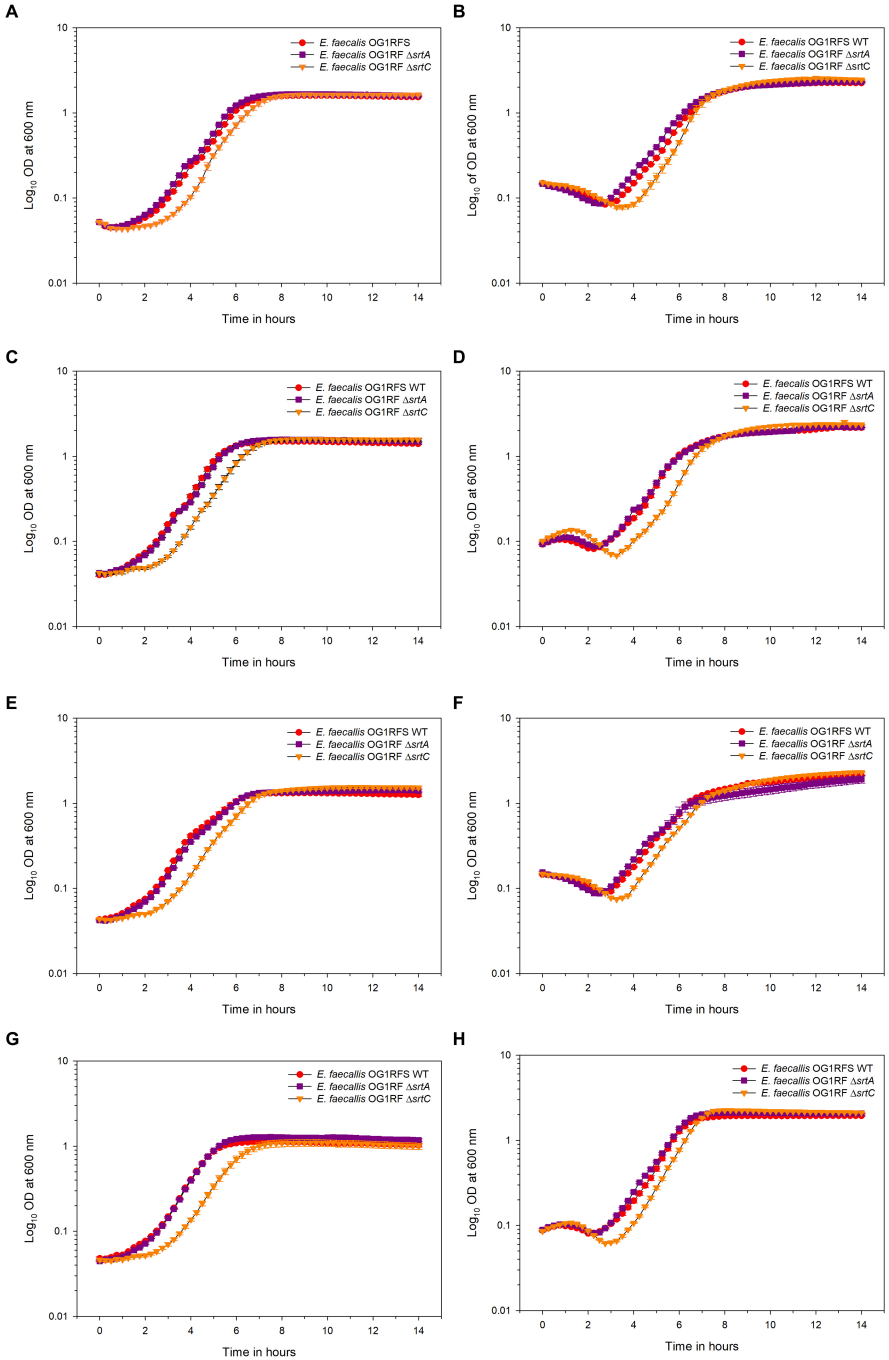
Growth of *E. faecalis* OG1RFS WT, *E. faecalis* Δ*srtA*, and *E. faecalis* Δ*srtC* in YP media supplemented with different carbohydrates at pH 7 and 10. **(A)** Growth of *E. faecalis* strains in YPD medium at pH 7; **(B)** Growth of *E. faecalis* strains in YPD medium at pH 10; **(C)** Growth of *E. faecalis* strains in YPS medium at pH 7; **(D)** Growth of *E. faecalis* strains in YPS medium at pH 10; **(E)** Growth of *E. faecalis* strains in YPL medium at pH 7; **(F)** Growth of *E. faecalis* strains in YPL medium at pH 10; **(G)** Growth of *E. faecalis* strains in YPM medium at pH 7; **(H)** Growth of *E. faecalis* strains in YPM medium at pH 10. Error bars represent the standard error of the means of OD_600_ at each time point.

To test the influence of pH on carbon source utilization, *E. faecalis* OG1RFS WT, *E. faecalis* OG1RF Δ*srtA* and *E. faecalis* OG1RF Δ*srtC* were incubated in YPD, YPS, YPL, and YPM at a pH of 7.4 and pH of 10 *in vitro*. No significant difference in the time required to reach mid-log phase growth was measured between *E. faecalis* OG1RFS WT and *E. faecalis* OG1RF Δ*srtA* in all media at pH 7.4. *E. faecalis* OG1RF Δ*srtC* lagged significantly behind *E. faecalis* OG1RFS in YPD (*p = 0.037), YPL (*p = 0.037), YPM (*p = 0.014), and behind *E. faecalis* OG1RF Δ*srtA* in YPD (*p = 0.037), YPL (*p = 0.021), and YPM (*p = 0.009) (Figure 3A). No significant differences in the time required to reach mid-log phase growth was measured between *E. faecalis* OG1RFS WT and *E. faecalis* OG1RF Δ*srtA* in all media at pH 10. *E. faecalis* OG1RF Δ*srtC* lagged behind *E. faecalis* OG1RFS in YPD (*p = 0.004), YPS (*p = 0.024), YPL (*p = 0.034), and behind *E. faecalis* OG1RF Δ*srtA* YPD (*p = 0.004), YPS (*p = 0.011), and YPL (*p = 0.006) (Figure 3B).

### 3.3 *Enterococcus faecalis* Δ*srtA* exhibits impaired persistence in the *Helicoverpa zea* GIT

**Figure 4:**
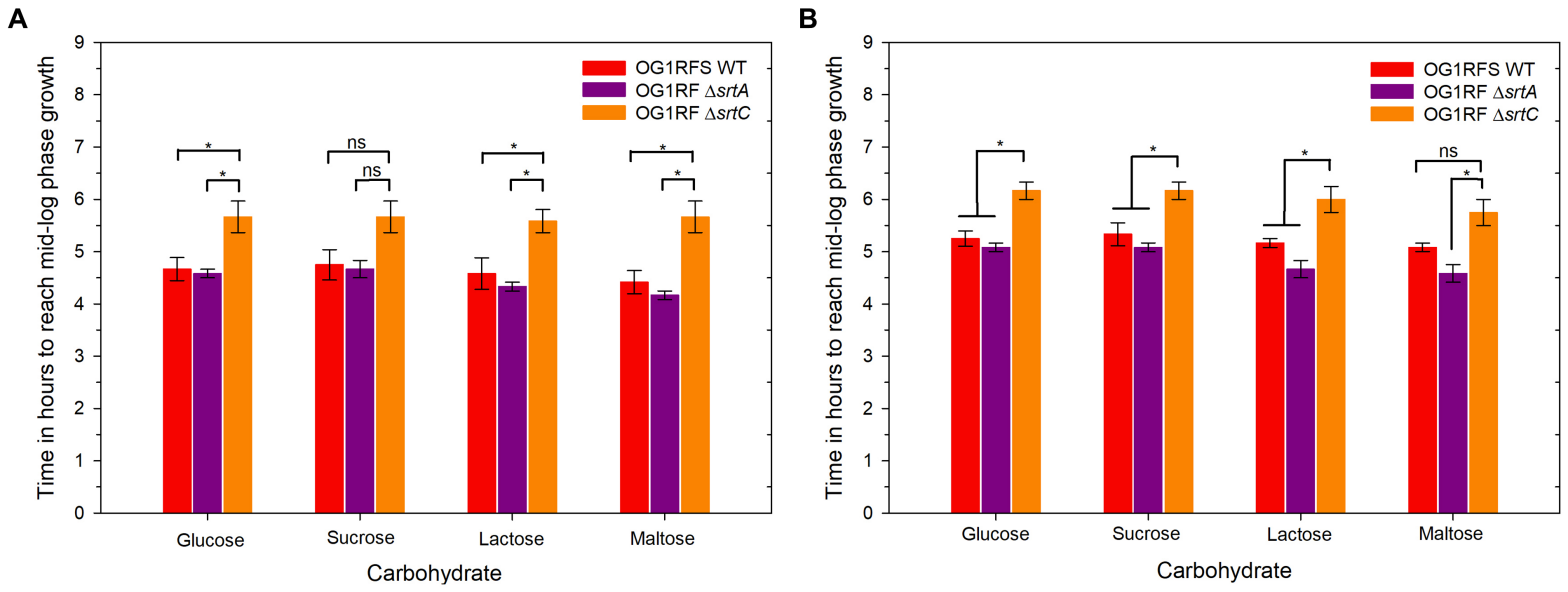
Elapsed time for *E. faecalis* OG1RFS WT, *E. faecalis* DsrtA, and *E. faecalis* D*srtC* to achieve mid-log phase growth in YP media supplemented with different carbohydrates at pH 7 and 10. **(A)** Time in hours for *E. faecalis* strains to reach mid-log phase growth in YPD, YPS, YPL, and YPM at pH 7; **(B)** Time in hours for *E. faecalis* strains to reach mid-log phase growth in YPD, YPS, YPL, and YPM at pH 10. Error bars represent the standard error of the means (*p < 0.05; Holm-Sidak, n.s. = not significant).

To test the role of *srtA* and *srtC* in mediating *E. faecalis* persistence in the *H. zea* gastrointestinal (GI) tract, we introduced *E. faecalis* OG1RFS WT, *E. faecalis* Δ*srtA*, and *E. faecalis* Δ*srtC* non-invasively in larval feeding bioassays. We recorded the weight of daily frass production and estimated the abundance of each strain in the larval GIT by serial diluting frass and plating the soluble fraction of homogenates on selective media. One day post-association, *H. zea* larvae fed *E. faecalis* OG1RF Δ*srtA* produced significantly more frass than larvae fed *E. faecalis* OG1RFS WT (*** *p* = 0.022, Cohen’s d = 0.26). On days two and three, there was no significant difference in the frass production of larvae fed either of the three strains (Figure 4A). No significant difference in the abundance of each strain in all larvae was detected on day one. However, the abundance of *E. faecalis* OG1RF Δ*srtA* in frass was significantly lower relative to *E. faecalis* OG1RFS WT on day two (Student’s *t*-test, *p* = 0.048, Cohen’s d = 0.92) and again on day three (Student’s t-test, *p* = 0.021, Cohen’s d = 1.04).

### 3.4 *Enterococcus faecalis* OG1RF Δ*srtA* and *Enterococcus faecalis* Δ*srtC* exhibit impaired biofilm formation

**Figure 5:**
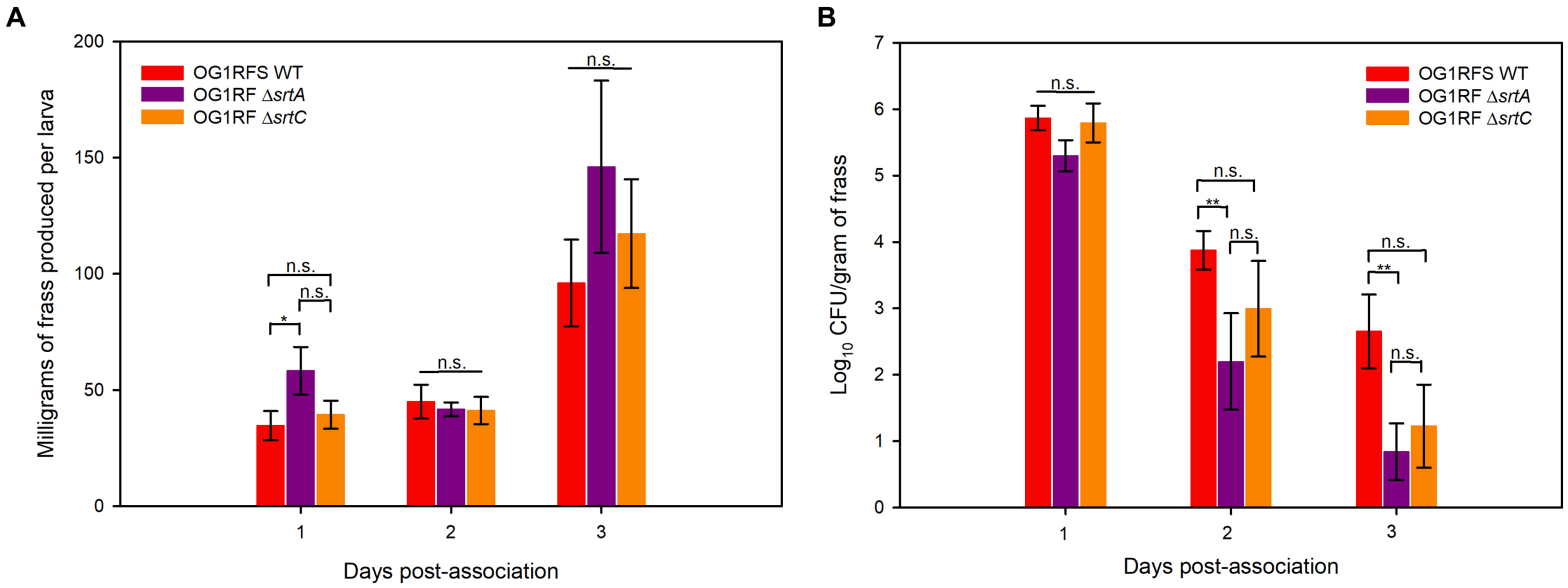
*H. zea* frass production and persistence of *E. faecalis* strains in the H. zea GIT. **(A)** Frass production by H. zea larvae associated with *E. faecalis* strains on day one; **(B)** Persistence of *E. faecalis* strains in the *H. zea* GIT following association on day one. Error bars represent the standard error of the means (*p < 0.05; Holm-Sidak, **p < 0.05; Welch’s test, n.s. = not significant).

The adherence of all three strains to the wells of polysulfone microtiter plates was tested in YPD at pH 7.4 and 10. *E. faecalis* OG1RFS WT early-stage biofilms were significantly larger (all ***p ≤ 0.006, all Cohen’s d ≥ 1.70) than those formed by *E. faecalis* OG1RF Δ*srtA* and Δ*srtC* in all YPD media at pH 7. Likewise, *E. faecalis* OG1RF Δ*srtA* early-stage biofilms were significantly larger (**p = 0.002, Cohen’s d = 1.54) than those formed by *E. faecalis* OG1RF Δ*srtC* (Figure 5A). In YPD media at pH 10, *E. faecalis* formed significantly larger early-stage biofilms than *E. faecalis* Δ*srtA* and Δ*srtC* (*** *p* ≤ 0.006, all Cohen’s d ≥ 1.42). *E. faecalis* OG1RF Δ*srtC* failed to form a detectable biofilm in YP but produced a significantly larger (**p = 0.041, Cohen’s d = 1.14) early-stage biofilm than *E. faecalis* OG1RF Δ*srtA* in YPD with 0.22% glucose. Biofilms formed in YPD with 0.67 and 2% glucose by *E. faecalis* OG1RF Δ*srtA* and *E. faecalis* Δ*srtC* were not significantly different (Figure 5B).

## 4 Discussion

Since the discovery of Sortase A in a *Staphylococcus aureus*, sortase-dependent proteins have been linked to diverse physiological processes in gram-positive bacteria, including adhesion and pilus assembly, evasion of the host immune system, virulence, and biofilm formation (Mazmanian et al., 1999; Kemp et al., 2007; Kline et al., 2009; Donahue et al., 2014; Call et al., 2015). While a majority of the data on sortases and sortase-dependent proteins has been generated using vertebrate models, their impact on the insect host-microbe relationship has not been investigated.

In the work presented here, we tested the impact of sortase deletions (Δ*srtA* and Δ*srtC*) on *E. faecalis* planktonic growth and biofilm formation *in vitro* under alkaline pH conditions similar to those encountered by microorganisms upon entry into the *H. zea* GIT. We also developed a non-destructive model for testing the role of bacterial genes that are essential to persistence in the GIT of lepidopteran larvae. The data suggest that Sortase A and Sortase C are essential for *E. faecalis* biofilm formation, but neither is essential for planktonic growth. In the *H. zea* model, we observed a significant persistence defect in the Δ*srtA* mutant relative to the WT OG1RFS control. Finally, the biofilm production of both Δ*srtA* and Δ*srtC* mutants was significantly reduced relative to the WT. We hypothesize that Sortase A- and Sortase C-dependent proteins mediate persistence in the *H. zea* GIT by binding to intestinal mucin and forming biofilms.

Requisite to developing a more accurate understanding of the commensal relationship between insect models and the stably colonized microorganisms in their GIT is the application of Rolf Freter’s nutrient-niche hypothesis which describes how the competition for nutrients (mostly polysaccharides) in the mucosal layer of intestinal environment drives the establishment of multispecies communities (Freter et al., 1983). The “restaurant” hypothesis of Tyrrell Conway and Paul Cohen builds on the nutrient-niche hypothesis by describing how *Escherichia coli* enters into mixed biofilms (“restaurants”) in the mucosal layer of the murine large intestine where obligate anaerobes release extracellular hydrolases that cleave complex polysaccharides in mucin to mono- and disaccharides which *E. coli* uses efficiently to colonize. In support of this hypothesis, *E. coli* strains MG1655 and Nissle 1917 were shown to reside in mixed biofilms in the mouse intestine (Leatham-Jensen et al., 2012; Adediran et al., 2014). Furthermore, expansion of *Salmonella enterica* serovar Typhimurium population in gnotobiotic mice was dependent on the release of fucose and sialic acid from mucosal polysaccharides by the obligate anaerobe, *Bacteroides thetaiotaomicron* (Ng et al., 2013).

A majority of the data on the spontaneous resistance of bacteria to streptomycin has pointed to single nucleotide polymorphisms in *rpsL*, the gene encoding the ribosomal S12 protein (Spagnolo et al., 2016; Shafipour et al., 2022). We isolated a streptomycin-resistant *E. faecalis* OG1RF clone and sequenced *rpsL* (Figure 1), and sequencing data indicated that the mutant harbors a nucleotide substitution which results in a lysine to arginine switch in the 56^th^ codon of the *rpsL* reading frame (See clone sequences in Supplementary material). During *in vitro* growth analyses, *E. faecalis* OG1RFS WT displayed greater than a 10-fold resistance to streptomycin levels routinely used for selection on agar plates (Figure 2).

**Figure 6:**
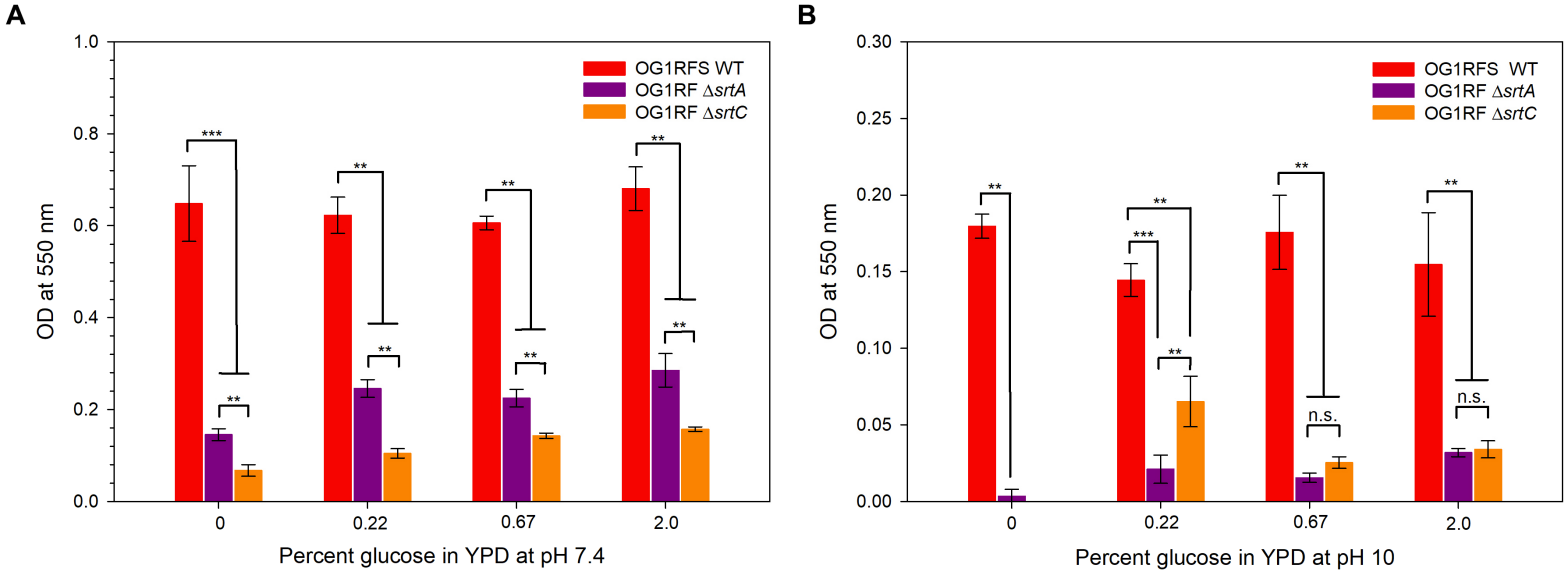
Biofilm formation by *E. faecalis* strains in YPD medium containing different concentrations of glucose. **(A)** Biofilm formation of *E. faecalis* strains in YPD media at pH 7; **(B)** Biofilm formation of *E. faecalis* strains in YPD media at pH 10. Error bars represent the standard error of the means (**p < 0.05; Welch’s test, ***p < 0.05; Mann-Whitney Rank Sum, n.s. = not significant).

Under physiological and alkaline planktonic growth conditions *in vitro*, the *E. faecalis* Δ*srtA* and Δ*srt*C mutants each grew to a final density similar to OG1RFS WT. However, the *E. faecalis* Δ*srtC* mutant displayed a significant delay in reaching mid-log phase growth relative to the WT and *ΔsrtA* strains (Figures 3, 6). In light previous reports which detected no significant difference in the *in vitro* growth of *E. faecalis ΔsrtA* and Δ*srtC* mutants relative to the WT strain, our observations may be an artifact of the components used in the preparation of the yeast peptone-based media (Kemp et al., 2007; Banla et al., 2019). The circumstances presented here, however, suggest a potential role exists for Sortase C-dependent proteins in signaling the initiation of exponential growth *in vitro* as well as compensatory signaling mechanisms which allow the Δ*srtC* mutant to recover. During increases in the environmental pH, *E. faecalis* experiences an influx of negatively charged hydroxide ions (OH^−^) which raises the internal pH. To acidify the cytoplasm and re-establish a pH gradient optimal for growth, it uses proton (H^+^) pump and potassium (K+) uptake systems and increases the expression of major heat shock proteins, DnaK and GroEL, which help prevent protein misfolding and degradation (Booth, 1985; Langer et al., 1992; Flahaut et al., 1997; Jain et al., 2017). In support of the former observation, we observed an immediate but temporary spike in the absorbance values of all *E. faecalis* strains following inoculation into BHI at pH 10. This suggests the uptake of OH^−^ into the cytoplasm lead to a temporary increase in the intracellular volume and not cell division (Figures 6B,D,F,H). In gnotobiotic mice, the obligate anaerobe, *Bacteroides thetaiotaomicron*, secretes polysaccharide hydrolases that digest complex polysaccharides in the mucosal layer as well as dietary fiber and cell debris. This releases fucose and sialic acid which, upon entry into the animal GIT, *Salmonella typhimurium* utilizes for growth (Ng et al., 2013). There is currently no data supporting a similar symbiotic relationship between obligate and facultative anaerobes in the insect GIT, nor a detailed description of the architecture of insect mucin to suggest the carbohydrate moieties that may potentially be released for bacteria growing planktonically. In a previous report it was demonstrated that mucus-forming mucins associated with the *Spodoptera frugiperda* peritrophic matrix (PM) immobilize digestive enzymes and aid in the digestion of substances prior to them traversing the PM into the ectoperitrophic space and that enhancins produced by *Trichoplusia ni* granulosis virus degraded intestinal mucins in the *Trichoplusia Ni* (cabbage looper) PM (Ferreira et al., 1994; Wang and Granados, 1997). These intestinal mucins are analogous to the MUC2 vertebrate mucin, the major constituent in the mucosal layer of the mammalian GIT (Atuma et al., 2001). The absence of information about the carbohydrate composition of mucins in the insect GIT presents challenges when determining how to model *E. faecalis* growth on different carbohydrates as an indication of its *in vivo* behavior. We tested *E. faecalis* utilization of four structurally different carbohydrates, three of which are reducing (glucose, lactose, and maltose) and one that is non-reducing (sucrose), provided as the most abundant carbohydrate in yeast-peptone supplemented media, to represent the flexibility of *E. faecalis* carbohydrate catabolism under alkaline conditions (Figure 4B). We hypothesize that *E. faecalis* persistence does not depend on planktonic growth alone, because the data do not provide evidence that the GIT population stabilized over the duration of the persistence bioassay.

Studies on the molecular biology underlying microbial colonization of the insect GIT have relied, almost exclusively, on destructive sampling methodologies, during which the sample population from which data is acquired changes as individuals are sacrificed (Holt et al., 2015; Teh et al., 2016; Zhang et al., 2022). However, the use of parallel experimental methodologies, data acquisition, and analysis will lead to an enhanced understanding of the molecular mechanisms utilized by microorganisms to colonize the insect GIT. We have established a non-destructive lepidopteran model for characterizing the role of bacterial genes that are essential to persistence in the GIT of lepidopteran larvae, The effects of sortase deletions exhibit bacterial species-, host-, and tissue-specificity, owing to the diverse roles performed by sortase-dependent cell surface proteins. For example, a *Streptococcus pneumoniae* Δ*srtA* mutant exhibited an *in vivo* colonization defect in competitive infections with its parental strain in a murine nasopharyngeal colonization model but showed no signs of attenuated virulence when injected intravenously (Paterson and Mitchell, 2006). In a murine model of renal abscess formation, all animals injected with a *Staphylococcus aureus* Δ*srtA* mutant successfully cleared the inoculum, while those injected with its parental strain developed kidney abscesses (Mazmanian et al., 2000). In our *H, zea* model, larvae fed the *E. faecalis* Δ*srtA* mutant produced significantly more frass relative to the WT strain one day post-association, but we measured no significant increase in frass production relative to the WT or the Δ*srtC* mutant on either day two or day three of the bioassay (Figure 4A). In previous reports, the *E. faecalis* LX10 population expanded ~5 log_10_ units in the *Bombyx mori* GIT between the first and fifth instars (Zhang et al., 2022), while both *E. faecalis* OG1RF (Holt et al., 2015) and *Enterococcus mundtii* (Teh et al., 2016) populations declined in the *Manduca sexta* (tobacco hornworm) and *Spodoptera littoralis* (cotton leafworm) GITs, respectively. Though we measured a significant difference in frass production by *H. zea* larvae fed the Δ*srtA* mutant one day post-association, the persistence of the Δ*srtA* and Δ*srtC* mutants in the *H. zea* GIT was lower than the WT strain two and three days post-association, while the Δ*srtA* mutant was significantly lower on both days relative to the WT strain (Figure 4B). Sortase A- and Sortase C-dependent proteins have been shown to mediate *E. faecalis* OG1RF persistence in a murine models of urinary tract infection and intestinal colonization, respectively (Kemp et al., 2007; Banla et al., 2019). Similarly, our data suggests that Sortase A-dependent proteins, which include the base pilin subunit (Ebp) of the endocarditis and biofilm-associated pilus polymerized by Sortase C, play a significant role in mediating the persistence of *E. faecalis*. Given the housekeeping role of Sortase A, it is plausible that the significant reduction in persistence of the Δ*srtA* mutant is due to its role in covalently attaching a larger number of proteins involved in adhesion to the peptidoglycan layer, relative to Sortase C, which specifically polymerizes the endocarditis- and biofilm-associated pilus following placement of Ebp by Sortase A.

The continuous flux of substances through the GITs of mammals and insects provides a continuous supply of nutrients that is difficult to replicate without a chemostat continuous culture system [reviewed in Bull (2010)]. Consequently, it is reasonable to assume that the static microtiter environment overestimates the extent to which biofilms form. It was demonstrated previously that an *E. faecalis* OG1RF Δ*srtC mutant* formed reduced biofilms on porcine mucin relative to *E. faecalis* OG1RF WT at physiological pH (Kemp et al., 2007). Accordingly, we measured statistically significant reductions in biofilms formed by Δ*srtA* and Δ*srtC* mutants relative to the WT strain at a pH of 7.4 and 10 (Figures 5A,B). At pH 7, the Δ*srtA* mutant formed larger biofilms than the Δ*srtC* mutant, an observation that was reversed at pH 10. These data suggest that Sortase C-dependent proteins play a larger role in mediating attachment to abiotic surfaces at physiological pH, whereas Sortase A-dependent proteins have a minimally larger role at pH 10. In light of the aforementioned limitations associated with assessing biofilm formation under static conditions, the significant reduction in biofilms formed by both mutants aligns with the results obtained from the persistence bioassay. Moreover, because *E. faecalis* has displayed high levels of resistance to the alkalinity of calcium hydroxide pastes and is the most frequently identified microbial species causing endodontic infections of the root canal space (Siqueira and de Uzeda, 1996; Gopikrishna et al., 2006), these results provide strong implications for the development of endodontic irrigants which exploit the mechanisms whereby sortase-dependent proteins, many of which remain uncharacterized (Banla et al., 2019), mediate invasion and growth in medicated canals.

There are key differences between the mammalian and insect GITs that should limit the extent to which results describing the molecular mediators of microbial persistence should be extrapolated. Most notably, the luminal pH of the mammalian and insect GITs maintain reported averages of 5.7–7.4 and 7–12, respectively, and the midgut region of the GIT in most insect is lined with the aforementioned PM, which is absent in mammals (Dow, 1992; Lehane, 1997; Fallingborg, 1999). Additionally, while sortases are responsible for the covalent anchoring of a large percentage of the proteins displayed on the surface of gram-positive bacteria, other non sortase-dependent proteins acting at or near the cell surface include lipoproteins and those which are attached by hydrophobic and/or electrostatic interactions [reviewed in Fischetti (2019)].

In conclusion, *E. faecalis* persistence in the *H. zea* GIT was neither strongly correlated with planktonic growth nor increased feeding and frass production following association (Figure 4). Interestingly, *s*canning electron microscopy and florescence *in situ* hybridization have identified biofilm-like structures formed by *Enterococcus* sp. *in* the GITs of *Hyles euphorbiae* (Spurge hawkmoth) and in the mucosal layer of the *Spodoptera littoralis* (cotton leafworm) GIT, respectively (Shao et al., 2014; Vilanova et al., 2016). Collectively these data suggest that *E. faecalis* SrtA and SrtC-dependent proteins may promote binding to intestinal mucins and biofilm formation which mediate persistence in the alkaline environment of the lepidopteran GIT.

## Data availability statement

The original contributions presented in the study are included in the article/Supplementary material. Further should be directed to JJ.

## Ethics statement

The manuscript presents research on animals that do not require ethical approval for their study.

## Author contributions

JJ: Conceptualization, Formal analysis, Investigation, Methodology, Resources, Supervision, Writing – original draft, Writing – review & editing. SH: Investigation, Methodology, Writing – review & editing. GB: Investigation, Methodology, Writing – review & editing.
