# Supplementary material for: Sortase-encoding genes, srtA and srtC, mediate Enterococcus faecalis OG1RF persistence in the Helicoverpa zea gastrointestinal tract
Source: Front Microbiol. 2024 Mar 18;15:1322303. doi: 10.3389/fmicb.2024.1322303 (PMC10982312; doi:10.3389/fmicb.2024.1322303)
Supplement: Supplementary file 1 [file Data_Sheet_1.docx]

| **Supplementary Table S1. Bacterial strains** | |  |  |  |  |  |
| --- | --- | --- | --- | --- | --- | --- |
|  |  |  |  |  |  |  |
| **Strains** | **Description^a^** | | | **Source** | | |
| OG1RF | Spontaneous Fus^R^, Rif^R^ wild-type strain | | | (Dunny et al., 1978) | | |
| OG1RFS | Spontaneous Str^R^ OG1RF | | | This study | | |
| OG1RF Δ*srtA* | *srtA* deletion mutant of OG1RF (IB10) | | | (Banla et al., 2019) | | |
| OG1RF Δ*srtC* | *srtC* deletion mutant of OG1RF (TX5470) | | | (Nallapareddy et al., 2006) | | |
|  |  |  |  |  |  |  |
| ^a^Fus= fusidic acid; Rif = rifampicin, Str = streptomycin, superscript "R" designates resistance | | | | | | |

**Supplemental References**

Banla, L.I., Pickrum, A.M., Hayward, M., Kristich, C.J., and Salzman, N.H. (2019). Sortase-Dependent Proteins Promote Gastrointestinal Colonization by Enterococci. *Infect Immun* 87.

Dunny, G.M., Brown, B.L., and Clewell, D.B. (1978). Induced cell aggregation and mating in Streptococcus faecalis: evidence for a bacterial sex pheromone. *Proc Natl Acad Sci U S A* 75**,** 3479-3483.

Nallapareddy, S.R., Singh, K.V., Sillanpaa, J., Garsin, D.A., Hook, M., Erlandsen, S.L., and Murray, B.E. (2006). Endocarditis and biofilm-associated pili of Enterococcus faecalis. *J Clin Invest* 116**,** 2799-2807.
